# *Ustilago maydis* produces itaconic acid via the unusual intermediate *trans*‐aconitate

**DOI:** 10.1111/1751-7915.12329

**Published:** 2015-12-07

**Authors:** Elena Geiser, Sandra K Przybilla, Alexandra Friedrich, Wolfgang Buckel, Nick Wierckx, Lars M Blank, Michael Bölker

**Affiliations:** ^1^ iAMB – Institute of Applied MicrobiologyABBt – Aachen Biology and BiotechnologyRWTH Aachen UniversityWorringerweg 1D‐52074AachenGermany; ^2^ Department of BiologyPhilipps‐University MarburgKarl‐von‐Frisch‐Straße 8D‐35032MarburgGermany; ^3^ LOEWE Center for Synthetic Microbiology (SYNMIKRO)Hans‐Meerwein‐StraßeD‐35032MarburgGermany

## Abstract

Itaconic acid is an important biomass‐derived chemical building block but has also recently been identified as a metabolite produced in mammals, which has antimicrobial activity. The biosynthetic pathway of itaconic acid has been elucidated in the ascomycetous fungus *A* *spergillus terreus* and in human macrophages. In both organisms itaconic acid is generated by decarboxylation of the tricarboxylic acid (TCA) cycle intermediate *cis*‐aconitate. Here, we show that the basidiomycetous fungus *U* *stilago maydis* uses an alternative pathway and produces itaconic acid via *trans*‐aconitate, the thermodynamically favoured isomer of *cis*‐aconitate. We have identified a gene cluster that contains all genes involved in itaconic acid formation. *Trans*‐aconitate is generated from *cis*‐aconitate by a cytosolic aconitate‐Δ‐isomerase (Adi1) that belongs to the PrpF family of proteins involved in bacterial propionate degradation. Decarboxylation of *trans*‐aconitate is catalyzed by a novel enzyme, *trans*‐aconitate decarboxylase (Tad1). Tad1 displays significant sequence similarity with bacterial 3‐carboxy‐*cis*,*cis*‐muconate lactonizing enzymes (CMLE). This suggests that *U* *. maydis* has evolved an alternative biosynthetic pathway for itaconate production using the toxic intermediate *trans*‐aconitate. Overexpression of a pathway‐specific transcription factor (Ria1) or a mitochondrial tricarboxylic acid transporter (Mtt1) resulted in a twofold increase in itaconate yield. Therefore, our findings offer new strategies for biotechnological production of this valuable biomass‐derived chemical.

## Introduction

**Figure 1:**
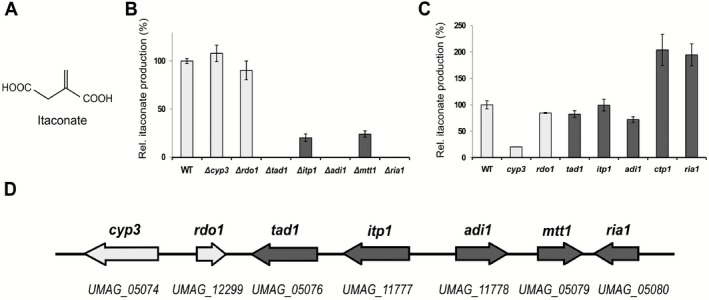
Gene cluster involved in itaconate biosynthesis. A. Chemical structure of itaconic acid. B. Deletion analysis of genes involved in itaconate formation in *U* *. maydis*. Itaconate levels in the supernatant were determined by HPLC after 96 h of cultivation. C. Relative itaconate production upon overexpression of individual cluster genes in *U* *. maydis* after 68 h in screening medium. Error bars indicate standard deviation of the mean (*n* = 2). D. Schematic overview of the itaconate biosynthesis gene cluster of *U* *. maydis*. Dark colour indicates genes directly involved in itaconate production and transport.

The unsaturated dicarboxylic acid itaconate (Fig. 1A) is commercially used in the production of pharmaceuticals, adhesives and as a copolymer for synthetic resins. Microbial itaconate production was first described for the ascomycetous fungus *Aspergillus itaconicus* (Kinoshita, 1932) and the related species *Aspergillus terreus* is still used for industrial production of itaconate via fermentation (Willke and Vorlop, 2001; Okabe *et al*., 2009; Klement and Büchs, 2013). Itaconic acid is also an interesting starting material for biofuel production because it can easily be converted into 3‐methyltetrahydrofuran (3‐MTHF), a fuel with excellent physical and chemical combustion properties (Geilen *et al*., 2010). Therefore, itaconic acid is regarded as one of the ‘top value added chemicals from biomass’ that can be subsequently converted to a number of high‐value chemicals or materials in the industry (Werpy and Petersen, 2004).

Quite recently, itaconate production by mammalian macrophages during inflammation has also been observed (Sugimoto *et al*., 2012; Michelucci *et al*., 2013; Cordes *et al*., 2015). There, it serves as an immune‐supportive metabolite that blocks the bacterial glyoxylate shunt by inhibition of isocitrate lyase (McFadden and Purohit, 1977). Numerous human pathogenic bacteria have evolved itaconate degradation and detoxification pathways as counter‐response, emphasizing the medical relevance for itaconate and its biochemistry (Sasikaran *et al*., 2014). For fungi, itaconate production is likely beneficial in an ecological context through several effects. In general, production of organic acids enables the liberation of micronutrients, such as phosphate and metals through chelating properties of the acids and a decrease of pH (Whitelaw, 1999; Landeweert *et al*., 2001). This pH decrease, combined with a high tolerance to low pH of the producing organism, provides a competitive advantage in carbon‐rich environments (Cray *et al*., 2013), complemented by the specific action of itaconate as inhibitor of isocitrate lyase (McFadden and Purohit, 1977).

A biosynthetic route for microbial itaconate formation via enzymatic decarboxylation of the tricarboxylic acid (TCA) cycle intermediate *cis*‐aconitate has been proposed already in 1957 (Bentley and Thiessen, 1957a, 1957b). The responsible genes, however, were identified only recently. The *A. terreus cadA* gene encodes a *cis*‐aconitate decarboxylase, which converts *cis*‐aconitate into itaconate (Dwiarti *et al*., 2002; Kanamasa *et al*., 2008). The *cadA* gene is part of a gene cluster that also comprises a mitochondrial TCA transporter, a membrane permease and a transcription factor proposed to regulate pathway‐specific expression of cluster genes (Li *et al*., 2011). The biochemical elucidation of itaconate production, including its genetic background, has led to a surge of metabolic engineering efforts in recent years with itaconate production established in several heterologous hosts (Kanamasa *et al*., 2008; Li *et al*., 2012; Blumhoff *et al*., 2013; Klement and Büchs, 2013; Blazeck *et al*., 2014; Huang *et al*., 2014; Okamoto *et al*., 2014; Chin *et al*., 2015; Vuoristo *et al*., 2015).

Itaconate production has also been observed in the phytopathogenic basidiomycete *Ustilago maydis* in a range of up to 45 g l^−1^ (Haskins *et al*., 1955; Guevarra and Tabuchi, 1990; Klement and Büchs, 2013; Maassen *et al*., 2014). *Ustilago maydis* serves as an important model organism due to its well‐characterized genome to study fungal virulence in plants (Kaemper *et al*., 2006; Brefort *et al*., 2009). In addition, the single‐celled (yeast‐like) morphology of this fungus in liquid media provides a major advantage in submerged fermentations by circumventing several problems of filamentous fungi, such as elevated viscosity and clogging, hindered oxygen transfer, sensitivity to hydro‐mechanical stress and laborious handling of spores (Klement *et al*., 2012). Therefore, we sought to investigate the itaconate biosynthesis pathway in *U. maydis*.

## Results

### Identification of an itaconate biosynthesis gene cluster in *U* *. maydis*

Genome analysis revealed that *U. maydis* contains no orthologue of the *A. terreus cis*‐aconitate decarboxylase CadA, which belongs to the PrpD family of proteins. This suggests that *U. maydis* uses an alternative biosynthetic route for itaconic acid production. We noticed that the *U. maydis* genome contains three genes (*UMAG_02807*, *UMAG_06058* and *UMAG_11778* ) coding for PrpF family proteins related to the 3‐methylitaconate‐Δ‐isomerase Mii. Mii converts methylitaconate into dimethylmaleate during nicotinate fermentation by *Eubacterium barkeri* and also shows activity towards itaconate (Velarde *et al*., 2009). Gene disruption experiments revealed that deletion of *UMAG_11778* abolished itaconate production (Fig. 1B and Supporting Information Fig. S1), pointing to an important role for this gene during itaconate biosynthesis. Interestingly, several neighbouring genes are co‐regulated with *UMAG_11778* during pathogenic development of *U. maydis* (Zheng *et al*., 2008). To test whether these genes also contribute to itaconate biosynthesis, we performed a systematic deletion analysis of the putative gene cluster (Fig. 1D). Itaconate production was completely abolished in mutants disrupted for *UMAG_05076*, *UMAG_11778* or *UMAG_05080*, whereas deletion of *UMAG_11777* and *UMAG_05079* resulted in decreased itaconate production (Fig. 1B). Deletion of the two leftmost genes of the potential gene cluster (*UMAG_05074* and *UMAG_12299*) did not affect itaconate production significantly (Fig. 1B), although they appear to be co‐regulated *in planta* (Zheng *et al*., 2008). The fully annotated sequence of the cluster is deposited in NCBI GenBank under accession number (*NCBI GenBank KT852988)*.

### Identification of the itaconate pathway‐specific regulator Ria1

Under itaconate producing conditions, transcription of all potential cluster genes with exception of *UMAG_05074* and *UMAG_12299* was at least 3.5‐fold induced (Supporting Information Fig. S2). *UMAG_05074* encodes a P450 monooxygenase of the CYP504 family and *UMAG_12299* a ring cleaving dioxygenase (Table 1)*.* Therefore, we named the respective genes *cyp3* and *rdo1*. *UMAG_05080* is predicted to encode a basic helix–loop–helix (bHLH) transcription factor (Table 1). Deletion of *UMAG_05080* resulted in significant reduction of expression (*UMAG_05079* 90%, *UMAG_11778* 85%, *UMAG_11777* 70%, *UMAG_05076*, *UMAG_05074* and *UMAG_12299* each 30%), whereas the overexpression of *UMAG_05080* was sufficient to trigger expression of most cluster genes (Supporting Information Fig. S3). Therefore, we assume that *UMAG_05080* encodes a pathway‐specific regulator of itaconate biosynthesis and renamed the corresponding gene *ria1* (regulator of itaconic acid).

**Table 1:** Itaconic acid biosynthesis gene cluster in *U* *. maydis.*

Gene (locus tag)	Putative function	Size (aa)	Closest homologue with known function or structure	*E*‐value	Signature pattern
*cyp3* (*UMAG_05074*)	Hydroxylation of itaconate	540	Phenylacetate hydroxylase (CYP504 family) *A. nidulans* (Mingot *et al*., 1999)	9e‐121	P450 Monooxygenase
*rdo1* (*UMAG_12299*)	unknown	180	Macrophage colonization factor *Salmonella enterica* (Shi *et al*., 2006)	1e‐37	Ring‐cleaving dioxygenase
*tad1* (*UMAG_05076*)	Decarboxylation of *trans*‐aconitate	493	3‐Carboxy‐*cis,cis*‐muconate lactonizing enzyme *Pseudomonas putida* (Yang *et al*., 2004)	3e‐101	3‐carboxy‐*cis,cis*‐muconate lactonizing enzyme (pCMLE)
*itp1* (*UMAG_11777*)	Export of itaconate	491	EmrD multi drug resistance *E. coli* (Yin *et al*., 2006)	2e‐11	Major facilitator superfamily (MFS)
*adi1* (*UMAG_11778*)	Isomerization of aconitate	443	Oxalo‐mesaconate tautomerase *Pseudomonas putida* (Nogales *et al*., 2011)	5e‐72	PrpF‐superfamily
*mtt1* (*UMAG_05079*)	Mitochondrial *cis*‐aconitate export	300	Citrate transport protein *S. cerevisiae* (Kaplan *et al*., 1995)	3e‐80	Mitochondrial carrier protein
*ria1* (*UMAG_05080*)	Regulator of itaconate biosynthesis genes	362	Upstream stimulatory factor 2 (USF2) (Gregor *et al*., 1990)	0.003	Helix–loop–helix transcriptional regulator

### *In vivo* characterization of itaconate biosynthesis genes

**Figure 2:**
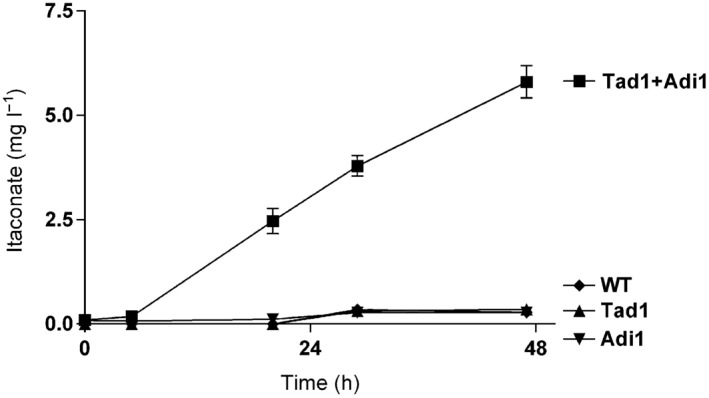
Heterologous itaconate production by *S* *. cerevisiae* expressing *U* *. maydis*' Adi1 and Tad1. Graph shows itaconate production over time by expression of the *U* *. maydis* itaconate biosynthetic enzymes Adi1 and Tad1 under control of the GAL4 promoter in the heterologous host *S* *. cerevisiae* in inducing medium (2% (w/v) galactose). Values are the arithmetic mean of two biological determinations. Error bars indicate standard deviation of the mean.

Two of the genes essential for itaconate biosynthesis (Fig. 1B) code for proteins with similarity to known enzymes (Table 1). The gene product of *UMAG_05076* is highly similar to prokaryotic 3‐carboxy‐*cis,cis*‐muconate lactonizing enzyme (CMLE) and the product of *UMAG_11778* belongs to the PrpF family of isomerases (Grimek and Escalante‐Semerena, 2004; Velarde *et al*., 2009; Nogales *et al*., 2011) (see Table 1). To test whether these two genes are directly responsible for itaconate biosynthesis, we expressed both in *Saccharomyces cerevisiae*. Although single expression of either gene did not trigger itaconate production, co‐expression of *UMAG_05076* and *UMAG_11778* resulted in itaconate production in this heterologous host (Fig. 2).

**Figure 3:**
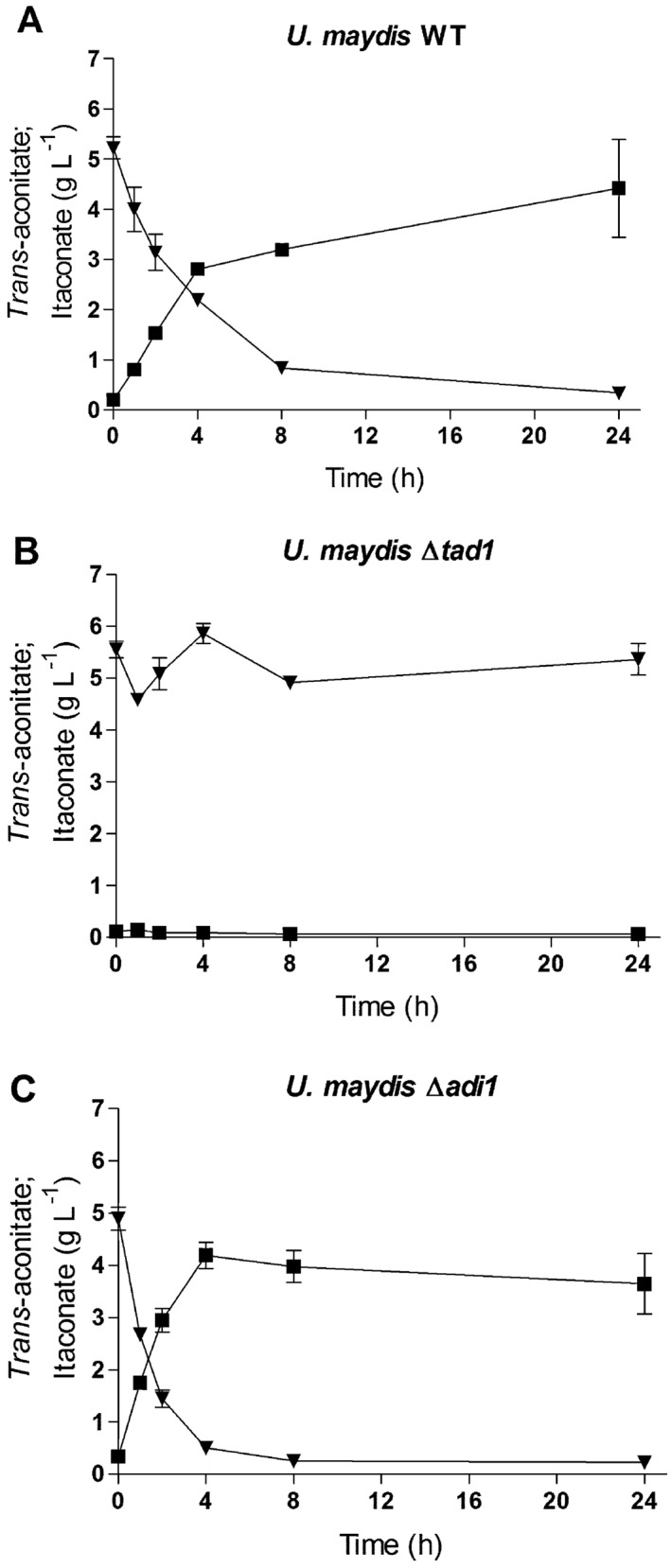
Permeabilized cell assay. Conversion of *trans*‐aconitate (▲) to itaconate (■) by permeabilized *U* *. maydis* cells. WT and mutant cells were incubated for indicated times after addition of 5 g l^−1^ of *trans*‐aconitate, (A) WT, (B) Δ*tad1* and (C) Δ*adi1*. Error bars indicate standard deviation of the mean (*n* = 2).

To elucidate the biosynthetic route of itaconate production in *U. maydis*, permeabilized cell assays were performed. Remarkably, itaconate formation was observed upon incubation of permeabilized cells with *trans*‐aconitate but not with *cis*‐aconitate, citrate or isocitrate (Fig. 3). Itaconate production from *trans*‐aconitate was not observed in Δ*UMAG_05076* cells, indicating that the catalytic activity of UMAG_05076 is required for itaconate formation.

### In vitro characterization of trans‐aconitate decarboxylase (UMAG_05076) and aconitate‐Δ‐isomerase (UMAG_11778)

**Figure 4:**
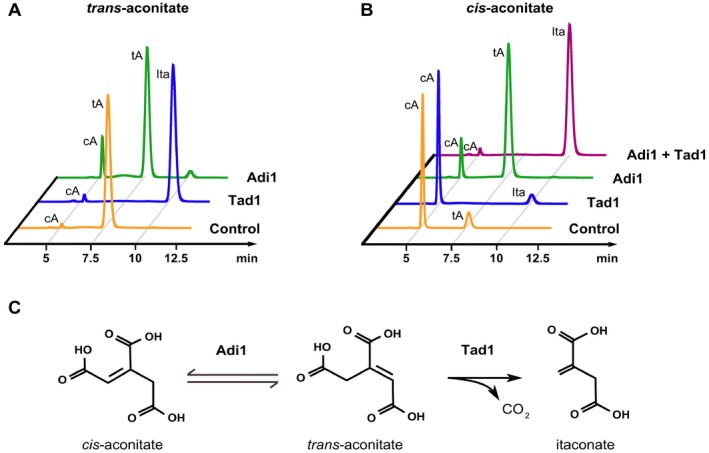
Enzymatic activities of Adi1 and Tad1. A. Conversion of *trans*‐aconitate (tA) into itaconate (Ita) or *cis*‐aconitate (cA) by purified Tad1 or Adi1. B. Conversion of *cis*‐aconitate into *trans*‐aconitate or itaconate by purified Tad1 and/or Adi1. Shown are HPLC chromatograms measured at 210 nm after 15 min of incubation with the indicated enzyme at room temperature. C. Schematic description of the itaconate biosynthesis pathway in *U* *. maydis*. Adi1 catalyzes isomerization of *cis*‐aconitate and *trans*‐aconitate. *Trans*‐aconitate is decarboxylated by Tad1 giving rise to itaconate.

In order to determine the catalytic activity of UMAG_05076, we heterologously expressed it as GST fusion protein in *E. coli* and tested the activity of the purified protein with different substrates *in vitro*. UMAG_05076 was able to convert *trans*‐aconitate completely into itaconate within 15 min (Fig. 4A), whereas *cis*‐aconitate was not converted (Fig. 4B). This indicates that the gene product of *UMAG_05076* acts as a decarboxylase specific for *trans*‐aconitate (Fig. 4C). Therefore, we termed the enzyme *trans*‐aconitate decarboxylase (Tad1) and renamed the corresponding gene *tad1*. To our knowledge, such enzymatic activity has not been described yet.

*Trans*‐aconitate is not a regular intermediate of cellular metabolism but acts as competitive inhibitor of the TCA cycle enzymes aconitase and fumarase (Saffran and Prado, 1949). We reasoned that during itaconate formation, *trans*‐aconitate might be generated from *cis*‐aconitate by the gene product of *UMAG_11778*. Indeed, recombinant GST‐UMAG_11778 fusion protein purified from *E. coli* was able to isomerize *cis*‐aconitate into *trans*‐aconitate in vitro (Fig. 4B). The enzyme catalyzed interconversion of *cis*‐aconitate and *trans*‐aconitate, resulting in equilibrium formation of 88% *trans* to 12% *cis*. Because we propose that *cis/trans*‐isomerization occurs via an allylic re‐arrangement (see below), we termed this enzyme aconitate‐Δ‐isomerase (Adi1) and renamed the respective gene *adi1*.

We were able to reconstitute the complete transformation of *cis*‐aconitate into itaconate *in vitro* upon incubation of *cis*‐aconitate with both Adi1 and Tad1 (Fig. 4B). This indicates that the coupled reaction appears to be irreversible. This may help to prevent any deleterious accumulation of the toxic intermediate *trans*‐aconitate during itaconate biosynthesis.

### Intracellular localization of the itaconate biosynthesis proteins

**Figure 5:**
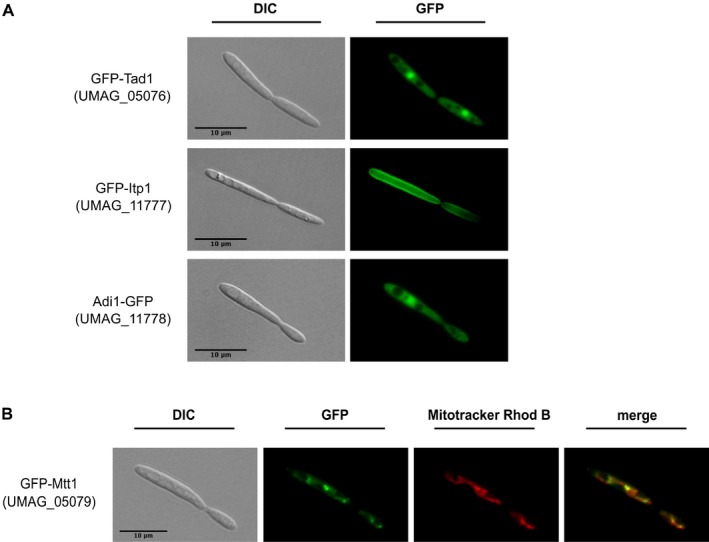
Intracellular localization of itaconate biosynthetic enzymes. A. Indicated GFP‐fusion proteins were analyzed by fluorescence microscopy. B. Mitochondrial localization of Mtt1 was confirmed by co‐localization with Mitotracker Rhodamine B (Mitotracker Rhod B). Scale bar = 10 μm.

**Figure 6:**
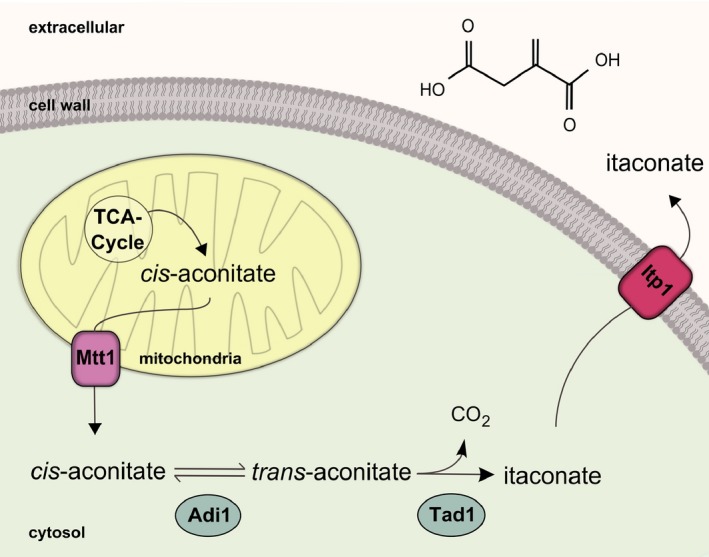
Proposed intracellular organization of the itaconate biosynthesis pathway in *U* *. maydis*. *Cis*‐aconitate is secreted by the mitochondrial tricarboxylate transporter Mtt1. In the cytosol *cis*‐aconitate is converted into itaconate via the intermediate *trans*‐aconitate. Secretion of itaconate into the medium is mediated by the major facilitator Itp1.

To determine the intracellular localization of the enzymes involved in itaconate biosynthesis, we followed green fluorescent protein (GFP) fusion proteins by fluorescence microscopy. Both Adi1 and Tad1 were located in the cytosol but were also found in the nucleus (Fig. 5A). The gene product of *UMAG_05079* is similar to mitochondrial TCA transporters (Table 1) and accumulated at distinct locations in mitochondria (Fig. 5B). UMAG_11777 is annotated as major facilitator superfamily (MFS) transporter (Table 1) and was found at the plasma membrane. Based on these data, we propose that the gene product of *UMAG_05079* actively exports *cis*‐aconitate from mitochondria into the cytosol. There it is converted into itaconate and secreted across the plasma membrane by the major facilitator UMAG_11777 (Fig. 6). Accordingly, we termed these transport proteins Mtt1 (mitochondrial TCA transporter) and Itp1 (itaconate transport protein) respectively. Taken together, our results demonstrate that *U. maydis* uses an alternative metabolic pathway to generate itaconate via the unusual intermediate *trans*‐aconitate.

### Improvement of itaconate production by overexpression of itaconate cluster genes

To test whether this newly gained knowledge of itaconate biosynthesis can be used for metabolic engineering of *U. maydis*, we overexpressed all cluster genes individually. More than a twofold increase in itaconate production was observed upon overexpression of either the pathway‐specific transcription factor Ria1, or the mitochondrial transporter Mtt1 (Fig. 1C). Surprisingly, overexpression of the P450 monooxygenase Cyp3 (Fig. 1C) caused a significant decrease in itaconate production to 20% of the wildtype level, most likely caused by P450‐dependent oxidation of itaconate (see Discussion).

## Discussion

So far, the molecular basis of itaconate biosynthesis has been elucidated only in the ascomycetous fungus *A. terreus* and in human macrophages (Kanamasa *et al*., 2008; Li *et al*., 2011; Michelucci *et al*., 2013). In both organisms, itaconate is generated by decarboxylation of *cis*‐aconitate. In *A. terreus*, the key enzyme CadA is part of a gene cluster that also contains mitochondrial and plasma membrane transporters and a transcription factor (Li *et al*., 2011). Here, we could show that in the basidiomycete *U. maydis* itaconate is produced by decarboxylation of *trans*‐aconitate. The critical enzyme Tad1 is remarkably different to *A. terreus* CadA. While CadA is related to pro‐ and eukaryotic methylcitrate‐dehydratases of the PrpD family, which catalyze an unusual syn‐elimination of water from (2S,3S)‐2‐methylcitrate, *U. maydis* Tad1 is highly similar to prokaryotic CMLE. Prokaryotic CMLE catalyze γ‐lactonization of 3‐carboxy‐*cis*,*cis*‐muconate during degradation of aromatic compounds (Harwood and Parales, 1996) via an anti‐1,2‐addition‐elimination reaction similar to that of class II fumarases (Williams *et al*., 1992). Therefore, we propose that Tad1 catalyzes decarboxylation of *trans*‐aconitate by addition of a proton to the double bond leading to transient formation of a carbenium ion and subsequent anti‐elimination of the carboxyl group (Supporting Information Fig. S4).

In *U. maydis*, *trans*‐aconitate is produced by isomerization of the TCA intermediate *cis*‐aconitate. We identified the aconitate‐Δ‐isomerase Adi1, which catalyzes interconversion of *cis*‐ and *trans*‐aconitate. Such isomerase activity has been described previously for plants that accumulate *trans*‐aconitate as antifeedant (Thompson *et al*., 1990) and for bacteria that are able to grow on *trans*‐aconitate (Altekar and Raghaven Rao, 1963). Although the responsible proteins have not been identified yet, biochemical studies in *Pseudomonas putida* indicated that isomerization proceeds via an allylic rearrangement (Klinman and Rose, 1971). The *U. maydis* aconitate isomerase Adi1 is closely related to methylitaconate‐Δ‐isomerase Mii from *E. barkeri* (Velarde *et al*., 2009) and to *Shewanella oneidensis* PrpF (Grimek and Escalante‐Semerena, 2004). For both enzymes, allylic mechanisms have been proposed. Therefore, we assume that Adi1 uses a similar mechanism to catalyse delta‐isomerization of *cis*‐aconitate into *trans*‐aconitate.

Overexpression of the P450 monooxygenase Cyp3 led to a drastic decrease of itaconate production, whereas deletion of *cyp3* resulted in a slight but not significant increase (*P* = 0.08). This suggests that itaconate might be a substrate for oxidation by Cyp3. We propose that Cyp3‐dependent oxidation results in the formation of 2‐hydroxyparaconate, which is a known byproduct of itaconate production in *Ustilago* species (Guevarra and Tabuchi, 1990). The presence of 2‐hydroxyparaconate, however, could not be confirmed yet because standards are not commercially available. The exact action and role of Cyp3 in relation to itaconate production will be studied in more detail in future experiments.

Overexpression of itaconate cluster genes *mtt1* and *ria1* resulted in significant improvement of itaconate yield. The effect of *ria1* is most probably due to direct upregulation of all cluster genes including *mtt1* (see Supporting Information Fig. S3). Together, this suggests that the putative mitochondrial *cis*‐aconitate transporter Mtt1 appears to be a bottleneck for itaconate production in *U. maydis*. There is an interesting analogy to fungal citrate production, where the ability of the mitochondrial citrate transporter to compete with the TCA cycle enzyme aconitase is critical for efficient citrate production (Karaffa and Kubicek, 2003).

In this manuscript, we describe a novel and alternative pathway for itaconate biosynthesis in *U. maydis* involving isomerization of *cis*‐aconitate into *trans*‐aconitate and its subsequent decarboxylation to generate itaconate. The molecular identification of Tad1 and Adi1 allows further biochemical and structural characterization of these interesting enzyme activities. In addition, our findings open up new avenues for metabolic engineering of homologous and heterologous itaconate production strains. Initial overexpression experiments already led to a twofold increase in itaconate production. Combinatorial overexpression of multiple genes will likely lead to further improvements, enabling to use *U. maydis* for sustainable large‐scale itaconate production.

## Experimental procedures

### Strains and culture conditions

*Escherichia coli* strain Top10 (Invitrogen) was used for all DNA manipulations. Cells were grown in dYT liquid medium (1% (w/v) yeast extract, 1.6% (w/v) tryptone, 0.5% (w/v) NaCl) at 37°C and maintained on dYT‐agar plates (dYT components + 1.3% (w/v) agar) at 4°C. *Ustilago maydis* strain MB215 (DSM17144) was used for all experiments. *U. maydis* cells were grown in YEPS medium (1% (w/v) yeast extract, 2% (w/v) peptone, 2% (w/v) sucrose) at 30°C and maintained on potato dextrose agar (PDA, Difco) at 4°C.

### Generation of deletion and overexpression strains

Deletion of single genes was performed by replacing the complete open reading frame (ORF) with a hygromycin resistance marker derived from plasmid pMF1‐H (Brachmann *et al*., 2004) by restriction with *SfiI*. Briefly, 1 kb recombination flanks were generated by polymerase chain reaction (PCR) using the primers listed in Supporting Information Table S1. Flanks, hygromycin‐cassette and vector pRS426 (Sikorski and Hieter, 1989) were assembled using homologous recombination in *S. cerevisiae*. Prior to transformation of *U. maydis*, the derived plasmid was restricted with *SspI* to isolate the deletion construct. Plasmid p123, which carries the strong constitutive *otef* promoter (Spellig *et al*., 1996) was used for generation of overexpression constructs by PCR and conventional cloning using primers indicated in Supporting Information Table S2. Plasmids were linearized with *SspI* prior to *U. maydis* transformation and all constructs were integrated into the *ip*‐locus by homologous recombination (Loubradou *et al*., 2001). Transformation of *U. maydis* was performed using standard protocols (Schulz *et al*., 1990). For selection of transformants, PDA plates with 200 μg ml^−1^ of hygromycin (Duchefa Biochemie) or 2 μg ml^−1^ of carboxin (Sigma‐Aldrich) were used. Correct integration of constructs was verified by Southern analysis or by PCR. For all deletion mutants itaconate production could be restored by complementation with the respective genes *in trans*, with the exception of deletion mutant *UMAG_05080*. This might be possibly related to lack of expression of this transcriptional regulator due to the ectopic insertion at the *ip*‐locus. For localization studies GFP‐fusion proteins generated in plasmid p123 were overexpressed in the respective deletion strains.

### Itaconate production procedure

Itaconate production cultures with *U. maydis* strains were performed in the System Duetz® (24 well plates) with a filling volume of 1.5 ml (shaking diameter = 50 mm, agitation speed = 300 r.p.m., temperature = 30°C, and relative air humidity = 80%) (Duetz *et al*., 2000). The screening medium contained 50 g l^−1^ of glucose, 0.8 g l^−1^ of NH_4_Cl, 0.2 g l^−1^ of MgSO_4_·7H_2_O, 0.01 g l^−1^ of FeSO_4_·7H_2_O, 0.5 g l^−1^ of KH_2_PO_4_, 33 g l^−1^, 1 ml l^−1^ of vitamin solution, 10 ml l^−1^ of trace element solution and as buffer 19.5 g l^−1^ 2‐(N‐morpholino)ethanesulfonic acid (MES) (Geiser *et al*., 2014). The pH of the MES stock solution was adjusted to 6.5 with NaOH. The vitamin solution contained (per litre) 0.05 g of D‐biotin, 1 g of D‐calcium panthotenate, 1 g of nicotinic acid, 25 g of myo‐inositol, 1 g of thiamine hydrochloride, 1 g of pyridoxol hydrochloride, and 0.2 g of para‐aminobenzoic acid. The trace element solution contained (per litre) 1.5 g of EDTA, 0.45 g of ZnSO_4_·7H_2_O, 0.10 g of MnCl_2_·4H_2_O, 0.03 g of CoCl_2_·6H_2_O, 0.03 g of CuSO_4_·5H_2_O, 0.04 g of Na_2_MoO_4_·2H_2_O, 0.45 g of CaCl_2_·2H_2_O, 0.3 g of FeSO_4_·7H_2_O, 0.10 g of H_3_BO_3_, and 0.01 g of KI.

Itaconate, citrate/isocitrate, *cis*‐aconitate and *trans*‐aconitate in the supernatants were analysed in a Beckmann Coulter System Gold High Performance Liquid Chromatography (Beckmann Coulter GmbH, Germany) with an Organic Acid Resin 300 × 8 mm column (CS‐Chromatography, Germany) and a differential refractometer LCD 201 (MELZ, Germany). As solvent, 5 mM H_2_SO_4_ with a flow rate of 0.6 ml min^−1^ and a temperature of 30°C was used. All samples were filtered with Rotilabo® syringe filters (CA, 0.20 μm, Ø 15 mm) and afterwards diluted 1:5 with 5 mM H_2_SO_4_. All relative components were identified via retention time and Ultraviolet (UV)/Refractive index (RI) quotient compared with corresponding standards. All values are the arithmetic mean of at least two biological replicates. Error bars indicate the deviation from the mean. Statistical analysis of significant difference was performed using Welch's test (level of significance of *P* ≤ 0.01).

### Permeabilized cell assay with *U* *. maydis* cells

The *U. maydis* strains were cultivated in 50 ml of screening medium in 500 ml Erlenmeyer flasks. Cells were grown for 24 h at 30°C and 250 r.p.m. Ten millilitres of culture broth of each strain was centrifuged (2200 × *g*, 5 min) and washed twice with 0.9% (w/v) sterile NaCl solution. Cells were re‐suspended in screening medium without carbon source. For permeabilization, cells were frozen and thawed twice. After addition of different substrate solutions (*trans*‐aconitate, *cis*‐aconitate, citrate and isocitrate) to a final concentration of 5 g l^−1^, samples of 900 μl were taken at different time points during incubation at 30°C. The enzymatic reaction was stopped by adding 100 μl of concentrated H_2_SO_4_. Substrate and product concentrations in the supernatants were analysed via high‐performance liquid chromatography (HPLC) as described before.

### Recombinant protein expression

Overexpression of GST fusion proteins UMAG_05076 (Tad1) and UMAG_11778 (Adi1) was performed in *E. coli* strain Rosetta 2 (DE3) (Novagen, Madison, WI, USA) using plasmids derived from pGEX4T‐1 (GE Healthcare, Waukesha, WI, USA). Plasmids were constructed via PCR and conventional cloning using primers listed in Supporting Information Table S3 and S4. Cells were pre‐cultivated in 200 ml of dYT medium to an OD_600_ of 0.6 at 37°C and protein production was induced by addition of 0.1 mM IPTG followed by overnight cultivation at 22°C. Cells were harvested by centrifugation, resuspended in lysis buffer (50 mM Tris‐HCl, pH 7.5, 100 mM NaCl, 10 mM MgCl_2_, 1 mM DTT, 1 mM PMSF) and lysed three times in a French pressure cell (max. 10.000 psi). After ultra‐centrifugation (1 h, 115 000 × *g*, 4°C), the supernatant was loaded on 500 μl of GSH Sepharose beads (Protino Glutathione Agarose 4B, Macherey‐Nagel) prior equilibrated with lysis buffer and incubated rolling at 4°C for 1 h. Beads were washed five times with lysis buffer (2 min, 700 × *g*, 4°C) and then eluted in 700 μl of elution buffer 30 min rolling at 4°C (100 mM Tris‐HCl, pH 9.0, 100 mM NaCl, 10 mM MgCl_2_, 1 mM DTT and 50 mM reduced glutathione). Protein concentration was determined using the Bradford test (Bradford, 1976).

### Enzyme activity assay

Ten micrograms of the corresponding purified protein was incubated with substrate (5 mM) for 15 min at room temperature in assay‐buffer (50 mM Tris‐HCl pH 7.5, 100 mM NaCl, 10 mM MgCl_2_, 1 mM DTT) in a final volume of 100 μl in a microtitreplate. The reaction was stopped by freezing samples at −20°C. Five microlitres reaction products were analysed by HPLC (Agilent Technologies 1200 Infinity series) at room temperature (22°C) with the following conditions. Column: organic acid resin, 250 × 8 mm (CS Chromatographie); detector: VWD 1260 (Agilent Technologies) at 210 nm; solvent: 2.5 mM sulfuric acid; flow rate: 1.0 ml min^–1^.

### Expression of itaconic acid biosynthesis genes in *S* *. cerevisiae*

Plasmids were constructed using the pGREG‐series published by Jansen *et al*., 2005 and the drag&drop‐cloning method described therein (Jansen *et al*., 2005) using primers listed in Supporting Information Table S5. Yeast strain ESM356 was used as host (Pereira *et al*., 2001). Transformants were pre‐cultured at 30°C and 250 r.p.m. overnight in 10 ml of SC dropout medium complemented with 2% (w/v) galactose. Main culture contained 50 ml of SC dropout medium complemented with 2% (w/v) galactose and cells from pre‐culture were diluted to a final OD_600_ of 0.5. Cultures were incubated for 48 h at 30°C and 250 r.p.m.. Itaconic acid production in the supernatant was detected by HPLC as previously described.

### RNA preparation from *U* *. maydis*, cDNA synthesis and reverse transcription polymerase chain reaction (qRT‐PCR)

Cells were grown under various conditions to determine gene expression. For itaconate‐producing conditions, cells were diluted from a pre‐culture to a final OD_600_ of 0.5 in screening medium (Geiser *et al*., 2014). After 12 h cells were harvested and diluted to a final OD_600_ of 0.8. Cells containing constructs with the arabinose‐inducible *crg*‐promoter were diluted from a pre‐culture to a final OD_600_ of 0.1 in minimal medium containing either 2% (w/v) glucose (control) or 2% (w/v) arabinose (induced) and harvested after 6 h. Cells grown in rich medium (YEPS) were diluted from a pre‐culture to a final OD_600_ of 0.1 and incubated for 6 h. All cultures were incubated at 30°C and 150 r.p.m. Cells were frozen in liquid nitrogen immediately after harvest.

For RNA extraction, cells were thawed and extracted using TRIzol (Chomczynski and Sacchi, 1987). After extraction, first‐strand cDNA synthesis kit (Thermo Scientific) was used to reverse‐transcribe 1 μg of total RNA to cDNA with oligo(dT) Primers. The amount of produced cDNA was determined using NanoDrop ND‐1000 (PEQLab). The qRT‐PCR analysis was performed using an iCycler (Bio‐Rad) using primers listed in Supporting Information Table S6. For the reaction Maxima SYBR Green/ROX qPCR Master Mix (Thermo Scientific) was used as recommended in the protocol. Gene expression levels were calculated relative to *ppi* expression levels using the cycle threshold (CT) 2^‐ΔCt^ method (Livak and Schmittgen, 2001).

Average expression and standard error of three independent experiments were calculated, and expression of the control (WT in YEPS) was set to 1. *P*‐values were estimated using an unpaired Student's *t*‐test. *P*‐values: *P < 0.05; **P < 0.01; ***P < 0.001.

### Microscopy

For microscopy, cells were incubated shaking overnight in 10 ml of YEPS at 30°C. Cells from pre‐culture were washed three times in water and diluted to a final OD_600_ of 0.2 in low fluorescent medium (YNB + 2% (w/v) glucose + 0.5% (w/v) (NH_4_)_2_SO_4_). Cells were grown to OD_600_ = 0.8 at 30°C and 150 r.p.m. For mitochondria staining 1 ml of cells at OD_600_ of 0.8 were harvested and re‐suspended in 10 mM Hepes pH 7.4 + 2% (w/v) glucose. Mitotracker Rhodamine B (1 μM in dimethylsulphoxide) was added to a final concentration of 0.1 μM and cells were incubated for 30 min at 30°C. Afterwards cells were washed three times in 10 mM Hepes pH 7.4. Cells were placed on agarose cushions and visualized by DIC and epifluorescence microscopy (GFP: 395 nm; Rhodamine: 500 nm) using a Zeiss Axiovert 200 microscope. Images were taken using a CCD camera (Hamamatsu Orca‐ER) with an exposure time of 30–300 ms. Image acquisition was performed using improvision volocity software and processing was done using imagej.

## Conflict of Interest

All authors have seen and approved the manuscript. All authors have contributed significantly to the work, and the manuscript has not been published and is not being considered for publication elsewhere. All authors declare that they have no competing financial interests.

## Author Contributions

E.G. and S.K.P. contributed equally to this manuscript. N.W., L.M.B., W.B. and M.B. conceived the project. N.W., L.M.B. and M.B. designed experiments and analysed results. M.B., S.K.P. and N.W. wrote the manuscript with the help of E.G. and L.M.B. S.K.P. generated *U. maydis*, *S. cerevisiae* and *E. coli* strains and performed enzyme activity assays and fluorescence microscopy. E.G. performed cultivation experiments, permeabilized cell assays and analysed itaconic acid production. A.F. and S.K.P. performed expression analysis of cluster genes.

## Supporting information


**Fig. S1.** A PrpF‐like protein is involved in itaconate formation.
**Fig. S2.** Expression of cluster genes depends on the pathway‐specific transcription factor Ria1.
**Fig. S3.** Induction of cluster genes by overexpression of Ria1.
**Fig. S4.** Hypothetical reaction mechanism of Tad1.

**Table S1.** Oligonucleotides for *U. maydis* gene deletion constructs.
**Table S2.** Oligonucleotides for *U. maydis* overexpression constructs.
**Table S3.** Oligonucleotides for recombinant protein expression in *E. coli.*
**Table S4.** Oligonucleotides for intron removal of UMAG_11778.
**Table S5.** Oligonucleotides for gene expression in *S. cerevisiae.*
**Table S6.** Oligonucleotides for quantitative RT‐PCR.
