# Supplementary material for: Ustilago maydis produces itaconic acid via the unusual intermediate trans‐aconitate
Source: Microb Biotechnol. 2015 Dec 7;9(1):116–26. doi: 10.1111/1751-7915.12329 (PMC4720413; doi:10.1111/1751-7915.12329)
Supplement: Supplementary file 1 — Fig. S1. A PrpF‐like protein is involved in itaconate formation. Fig. S2. Expression of cluster genes depends on the pathway‐specific transcription factor Ria1. Fig. S3. Induction of cluster genes by overexpression of Ria1. Fig. S4. Hypothetical reaction mechanism of Tad1. [file MBT2-9-116-s001.docx]

**Supporting Information Figures**

**FIGURE S1. A PrpF-like protein is involved in itaconate formation.** Itaconate concentration was determined in *U. maydis* PrpF deletion mutants after cultivation for 96 h in screening medium. Values are given as relative concentrations compared to the wildtype (in %). Error bars indicate standard deviation of the mean (n=2).


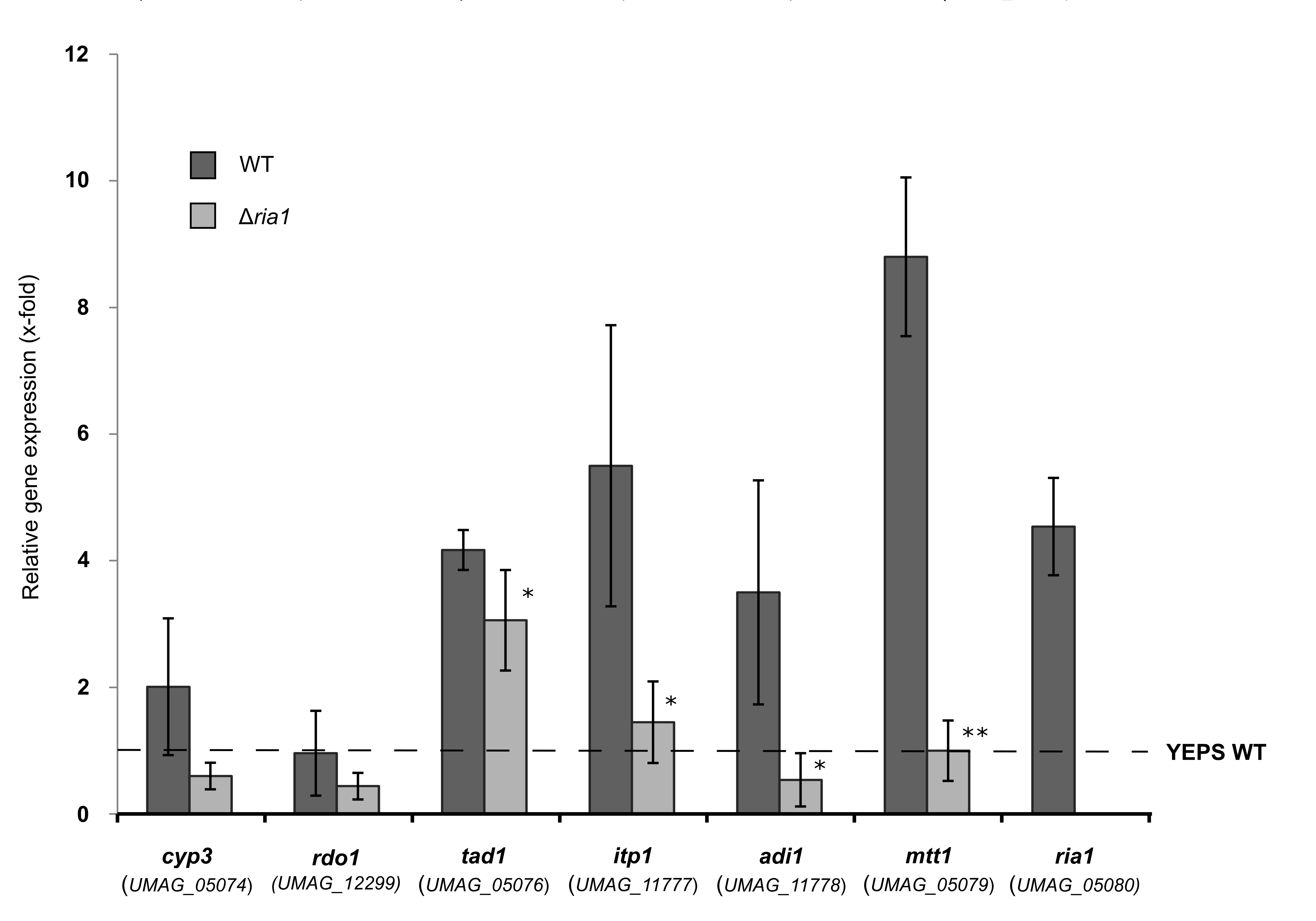


**FIGURE S2. Expression of cluster genes depends on the pathway-specific transcription factor Ria1.** Relative expression of cluster genes under itaconate producing conditions were determined for *U. maydis* WT and ∆*ria1* mutants by qRT-PCR after 12 h in screening medium (SM). Error bars indicate standard deviation of the mean (n=3). P-values: <0.05 = *; <0.01 = **; <0.001 = ***

**
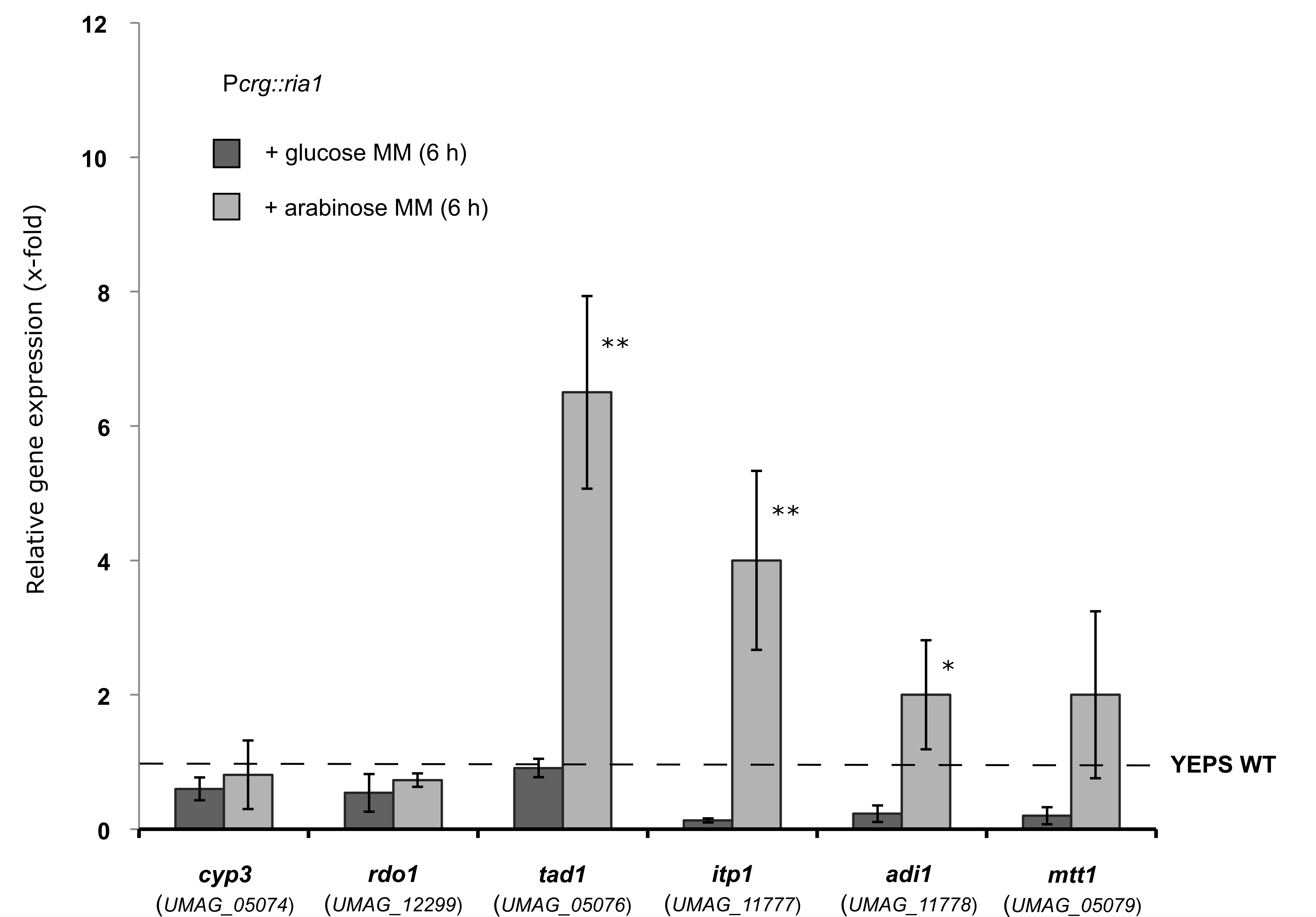
**

**FIGURE S3. Induction of cluster genes by overexpression of Ria1.** Relative expression of cluster genes was determined after overexpression of *ria1* under control of the arabinose-inducible promoter *crg1*. Cells were grown in minimal medium (MM) with glucose (2 %) and shifted for 6 hours to arabinose (2 %) or glucose (2 %), respectively. Error bars indicate standard deviation of the mean (n=3). P-values: <0.05 = *; <0.01 = **; <0.001 = ***


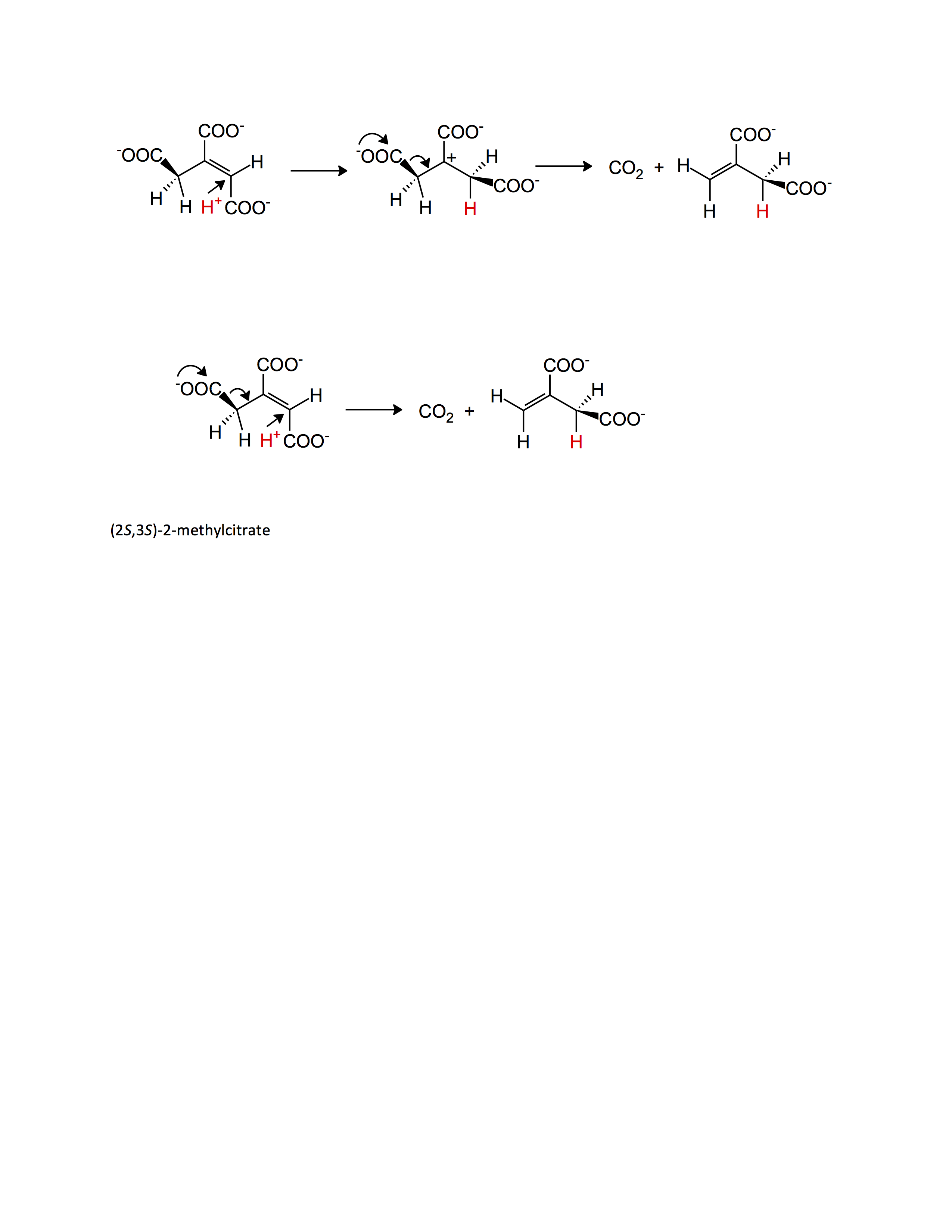


**FIGURE S4. Hypothetical reaction mechanism of Tad1.** The CMLE-like *trans*-aconitate decarboxlase Tad1 is proposed to catalyze an anti-1,2-addition-elimination reaction involving a transient carbenium-ion.
