# Supplementary material for: Ustilago maydis produces itaconic acid via the unusual intermediate trans‐aconitate
Source: Microb Biotechnol. 2015 Dec 7;9(1):116–26. doi: 10.1111/1751-7915.12329 (PMC4720413; doi:10.1111/1751-7915.12329)
Supplement: Supplementary file 2 — Table S1. Oligonucleotides for U. maydis gene deletion constructs. Table S2. Oligonucleotides for U. maydis overexpression constructs. Table S3. Oligonucleotides for recombinant protein expression in E. coli. Table S4. Oligonucleotides for intron removal of UMAG_11778. Table S5. Oligonucleotides for gene expression in S. cerevisiae. Table S6. Oligonucleotides for quantitative RT‐PCR. [file MBT2-9-116-s002.docx]

**Supporting Information Table 1:** Oligonucleotides for *U. maydis* gene deletion constructs

| **Name^a^** | **Sequence (5´->3´)** |
| --- | --- |
| LF fwd 05074 | gtaacgccagggttttccccagtcacgacgaatattccacccgcttggcagctccagttgg |
| LF rev 05074 | ccgcaattgtcacgccatggtggccatctaggcctgcgacatacaaca |
| RF fwd 05074 | gcgccgcgaagctgtgcggccgcattaataggcctgagtggcctcgcctcgcctctccgctcgctg |
| RF rev 05074 | gcggataacaatttcacacaggaaacagcaatattccgcaacgcagtcgtaacacgatcttg |
| LF fwd 05076 | gtaacgccagggttttcccagtcacgacgaatattacatacgtgatgccaatacatttcc |
| LF rev 05076 | gcggccgcaattgtcacgccatggtggccatctaggccactggatcgacgtaaaagccagccgg |
| RF fwd 05076 | gcgaagctgtgcggccgcattaataggcctgagtggcctacgtgtaccttgttggcctccctcg |
| RF rev 05076 | gcggataacaatttcacacaggaaacagcaatattgacttttacacgcgcctcggc |
| LF fwd 05079 | gtaacgccagggttttcccagtcacgacgaatatttcgatcgagcatccgagcggcg |
| LF rev 05079 | gcggccgcaattgtcacgccatggtggccatctaggccggtggatgacgaatctcaagagcg |
| RF fwd 05079 | gcgaagctgtgcggccgcattaataggcctgagtggccttagctgtgccccctcgcgtgtttcc |
| RF rev 05079 | gcggataacaatttcacacaggaaacagcaatattctgctccagcaggcgtgcgacg |
| LF fwd 05080 | gtaacgccagggttttcccagtcacgacgaatattgttgactgcggactgcggactgc |
| LF rev 05080 | gcggccgcaattgtcacgccatggtggccatctaggccgttggcaggacgctcgtcgtcgc |
| RF fwd 05080 | gcgaagctgtgcggccgcattaataggcctgagtggcccgtgtcaagtcgaatccgtgattctc |
| RF rev 05080 | gcggataacaatttcacacaggaaacagcaatattgtccaactcgagcaactaccttgccgg |
| LF fwd 11777 | gtaacgccagggttttcccagtcacgacgaatattccccattttcacgtttgttac |
| LF rev 11777 | gcggccgcaattgtcacgccatggtggccatctaggccttcgacttggtggatactgcggc |
| RF fwd 11777 | gcgaagctgtgcggccgcattaataggcctgagtggcctagcagagacgatggccaccttggagg |
| RF rev 11777 | cgcagacaaggaagcgtcgtacaatattgctgtttcctgtgtgaaattgttatccgc |
| LF fwd 11778 | gcaatatttcaccgttgttgggtcg |
| LF rev 11778 | atggcctgagtggccgagctgggtcgagtcaagacc |
| RF fwd 11778 | atggccatctaggcctgttttgcgtcttggatgc |
| RF rev 11778 | gcaatattcccgtgcacatcgcgcg |
| LF fwd 12299 | gtaacgccagggttttcccagtcacgacgaatattgatgctgcgatctccgggcag |
| LF rev 12299 | cgagtgcggccgcaattgtcacgccatggtggccatctaggccgtgtggtggtctgtgaatcaagtcgtg |
| RF fwd 12299 | ccgcgaagctgtgcggccgcattaataggcctgagtggccacgctccaacaaatctcctgagttcagc |
| RF rev 12299 | gcggataacaatttcacacaggaaacagcaatattgcgtggcacacggaacacgaccgc |

a: LF: left flank. RF: right flank.

**Supporting Information Table 2:** Oligonucleotides for *U. maydis* overexpression constructs

| **Name** | **Sequence (5´->3´)** |
| --- | --- |
| 05074 fwd | gtacccgggatgaacacgaccaaactactcggtaccg |
| 05074 rev | ctagcggccgcctattgtttgtgagcacgggggaaag |
| 05076 fwd | gtcggatccatggcacctgcactcaacgcaaaccctacc |
| 05076 rev | cgggcggccgctcaggccgacgatgggcgagacagc |
| 05079 fwd | gtcggatccatgccgccgtctggccgtaaagtgtcg |
| 05079 rev | cgggcggccgcctacgactcgggacctgctaggagccag |
| 05080 fwd | atcggatccatgcgctttgcagggatgagttgcg |
| 05080 rev | tcagcggccgcttatcggtgacgtttcttggacctgg |
| 11777 fwd | gtcggatccatggaccaagccgatcattccggcg |
| 11777 rev | cgggcggccgctcacgagtgtttgcgagctgcccac |
| 11778 fwd | gtcggatccatgttgcatccgatcgataccacc |
| 11778 rev | cgggcggccgcctaagagaggctgcggtcggatgc |
| 12299 fwd | ctgcccgggatgttgcgttctagccaggccag |
| 12299 rev | tcagcggccgctcactttgcgtcttggtacgacgag |

**Supporting Information Table 3:** Oligonucleotides for recombinant protein expression in *E. coli*

| **Name** | **Sequence (5´->3´)** |
| --- | --- |
| 05076 fwd | cgtggatccatggcacctgcactcaacgcaaac |
| 05076 rev | cgggaattctcaggccgacgatgggcgagacagc |
| 11778 fwd | cagggatccatgttgcatccgatcgatacc |
| 11778 rev | tcggaattcctaagagaggctgcggtcgg |

**Supporting Information Table 4:** Oligonucleotides for intron removal of UM_11778

| **Name** | **Sequence (5´->3´)** |
| --- | --- |
| 11778 A fwd | cagggatccgatgttgcatccgatcgatacc |
| 11778 A rev | gtgacaagcctttggcgtaaaatagcgtgcggtaaacgtggtcg |
| 11778 B fwd | attttacgccaaaggcttgtcacaacgc |
| 11778 B rev | tcggaattcctaagagaggctgcggtcgg |

**Supporting Information Table 5:** Oligonucleotides for gene expression in *S. cerevisiae*

| **Name** | **Sequence (5´->3´)** |
| --- | --- |
| 05076 fwd | gaattcgatatcaagcttatcgataccgtcgacaatggcacctgcactcaacgcaaaccc |
| 05076 rev | gcgtgacataactaattacatgactcgaggtcgactcaggccgacgatgggcgagacagc |
| 11778 fwd | gaattcgatatcaagcttatcgataccgtcgacaatgttgcatccgatcgatacc |
| 11778 rev | gcgtgacataactaattacatgactcgaggtcgacctaagagaggctgcggtcggatgcg |

**Supporting Information Table 6:** Oligonucleotides for quantitative RT-PCR

| **Name** | **Sequence (5´->3´)** | **Length (bp)** | **Melting Temp. (°C)** | **Product Size (bp)** |
| --- | --- | --- | --- | --- |
| RT 05074 fwd | gatttgctaacttggcgctc | 20 | 60 |  |
| RT 05074 rev | ccttgctctttgcattagcc | 20 | 60 | 111 |
| RT 05076 fwd | caatgcaacacaacctggac | 20 | 60 |  |
| RT 05076 rev | gagtttttcaagctcggtgc | 20 | 60 | 212 |
| RT 05079 fwd | tatctccgatcgaggtggtc | 20 | 60 |  |
| RT 05079 rev | tgttcttgagcatactgcgg | 20 | 60 | 150 |
| RT 05080 fwd | gcgttacagaccgaaaccat | 20 | 60 |  |
| RT 05080 rev | atcggtgacgtttcttggac | 20 | 60 | 242 |
| RT 11777 fwd | tgaaacgcttcgagtcattg | 20 | 60 |  |
| RT 11777 rev | tgcttgaacagatacggcag | 20 | 60 | 267 |
| RT 11778 fwd | ttcgttgacctccaaggtg | 20 | 60 |  |
| RT 11778 rev | gacctcgttcaatggcaaat | 20 | 60 | 168 |
| RT 12299 fwd | tgtttgtctgcttggtctcg | 20 | 60 |  |
| RT 12299 rev | tcttgcaggtgtttctgtcg | 20 | 60 | 282 |
